# Altruistic food sharing behavior by human infants after a hunger manipulation

**DOI:** 10.1038/s41598-020-58645-9

**Published:** 2020-02-04

**Authors:** Rodolfo Cortes Barragan, Rechele Brooks, Andrew N. Meltzoff

**Affiliations:** 1 Institute for Learning & Brain Sciences, University of Washington, Seattle, USA; 2 Department of Psychology, University of Washington, Seattle, USA

**Keywords:** Human behaviour, Psychology

## Abstract

Altruistic behavior entails giving valuable benefits to others while incurring a personal cost. A distinctively human form of altruistic behavior involves handing nutritious food to needy strangers, even when one desires the food. Engaging in altruistic food transfer, instead of keeping the food, is costly, because it reduces the caloric intake of the benefactor vis-à-vis the beneficiary. Human adults engage in this form of altruistic behavior during times of war and famine, when giving food to others threatens one’s own survival. Our closest living primate relatives, chimpanzees (*Pan troglodytes*) and bonobos (*Pan paniscus*), exhibit notable constraints on the proclivity to engage in such food transfer (particularly chimpanzees), although they share many social-cognitive commonalities with humans. Here we show that in a nonverbal test, 19-month-old human infants repeatedly and spontaneously transferred high-value, nutritious natural food to a stranger (Experiment 1) and more critically, did so after an experimental manipulation that imposed a feeding delay (Experiment 2), which increased their own motivation to eat the food. Social experience variables moderated the expression of this infant altruistic behavior, suggesting malleability.

## Introduction

Human adults engage in types of altruistic behavior not seen in other animals^1–3^. For example, adults give away precious resources to strangers, even when the cost is considerable^4^. A particularly meaningful category of resources involves caloric benefits—food. Human adults, both in hunter-gatherer and industrialized contexts^5,6^, have developed customs and institutions to provide needy people with food even when it is scarce and the donator needs the food themselves^7^. This altruistic response toward those in want of food has not been demonstrated by chimpanzees, even though this topic has attracted a great deal of study using a wide range of different paradigms^1,8–13^. Based on a comprehensive review of field and laboratory studies, evolutionary biologists have suggested that episodes of food sharing among chimpanzees are “best regarded as a passive process of selective relinquishment” (p. 452)^8^ and that “voluntary handing over of food is virtually absent” (p. 434)^8^. Although chimpanzees hand over common objects^9^, they do not use this ability to actively transfer nutritious, calorie-rich natural foods such as bananas^10^. The sociality profile of bonobos differs from chimpanzees^14,15^. Bonobos share food under many circumstances^14–19^ (Supplementary Information Note 1), but it is not clear how readily they hand over immediately edible, high-value food items in their possession (e.g., a piece of fruit, cracked nuts) rather than eating it themselves. In light of these patterns, we sought to test whether human infants readily and repeatedly engage in altruistic food transfer to a stranger, and do so when their favorite foods are involved and the motivation for eating the food is induced through an experimental manipulation (Experiment 2). We used a nonverbal, out-of-reach object test^9,15^, which in the future could be adapted and applied with non-human primates.

Alongside the idea that altruistic food transfer occurs more readily among humans than non-human primates, prior work has examined possible social and cultural contributors to the expression of altruistic helping behavior in human adults^20–22^ and children^23–25^. A leading hypothesis from social psychology is that families from different cultures tend to vary in the value they place on being a harmonious, empathic, and “other-regarding” group member^26,27^—and this may be associated with childrearing practices that support the expression of altruistic behavior^26,28^. Prior work has also suggested that sibling experience provides a context for the development of cooperative skills^29^. Thus, we explored whether the expression of human infants’ altruistic food transfer behavior to a stranger is related to these types of prior experiences.

Our investigation experimentally tested whether human infants, at 19 months of age, in the absence of any verbal request, spontaneously, repeatedly, and swiftly give away desirable food to a begging stranger (Experiment 1). Moreover, we tested this idea under evolutionarily relevant conditions designed to increase hunger (Experiment 2). Our current investigation used a design that builds upon and goes beyond related investigations with infants^9,25,30–35^ (Supplementary Information Note 2). It does so inasmuch as we focused on investigating infant spontaneous food sharing under conditions in which: (i) we did not use explicit verbal requests or prompts to elicit infants’ helping, (ii) we used nutritious, highly desirable natural food (bananas, grapes, etc.) rather than manufactured food, and (iii) we designed Experiment 2 to increase infants’ desire for the food by testing infants when the parents thought their infant would be hungry (immediately prior to their next feeding).

In two experiments we used a free-ranging human infant paradigm to assess infants’ offering of nutrient-dense food (a piece of banana, a blueberry, half a grape, and a piece of strawberry). The fruits, evolutionarily significant as a food group due to their nutrients and their sweetness^8,10^, were freshly cut prior to the experiments, such that infants could see, feel, and smell the fruits when they approached and touched them. Previous tests specifically showed that chimpanzees do not transfer these four high-energy fruits to conspecifics^10^ and data from our research (below) showed that they were favorite foods of human infants at this age.

Infants randomly assigned to the “Begging Experimenter Group” (experimental group) witnessed an experimenter pick up and fumble each of the fruits, accidently dropping them onto a tray. The experimenter tried to reach for the dropped fruit with his outstretched arm (seemingly begging for the food), but he could not attain the food, because he was blocked by the table between him and the tray. Infants randomly assigned to the “Non-Begging Experimenter Group” (control group) witnessed the same experimenter intentionally throwing each of the fruits onto the tray and looking at the fruit without attempting to reach it. These procedures and controls were based on classic studies of chimpanzees^9^ who, when subjected to these experimental and control procedures, were shown to hand over objects of no metabolic importance (sponge, tongs, and other nonedibles) to a human experimenter who had accidently dropped them, confirming that primates possess the requisite skills to transfer objects to non-kin others, even though they are not reported to do so in the case of highly desirable food^1,9^. In the current studies, the same experimenter transferred the same fruits to the same tray in both the experimental and the control groups—thus the starting state (fruit-in-adult’s-hand) and the end-state (fruit-in-floor-tray) was equated for both the experimental and control groups. If infants were simply motivated to return food to the adult who originally had it, transfers would be evident in the control group (because in both the experimental and control groups, the adult started off with the fruit in his hand). In the current studies, the experimenter did not express any displeasure and presented a neutral facial expression throughout the test trials regardless of infants’ responses.

## Results

### Experiment 1

**Figure 1:**
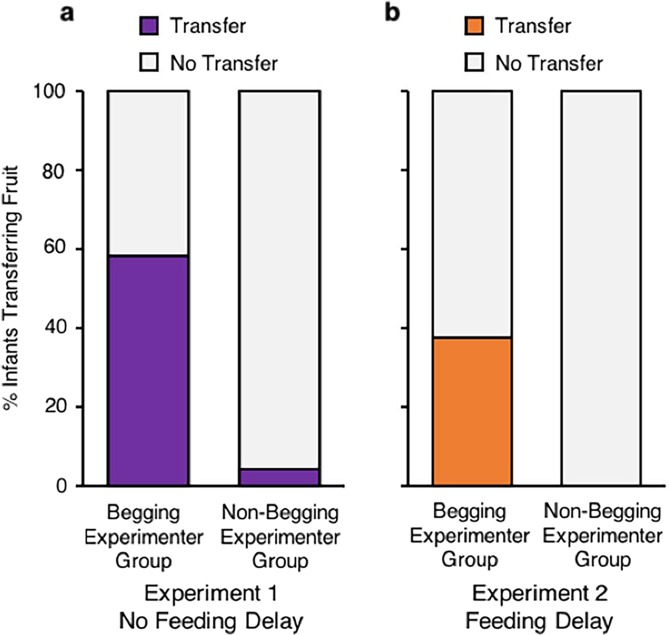
Percent of infants in each group transferring fruit. **(a)** Results for Experiment 1. **(b)** Results for Experiment 2.

Each child was given the four trials, one for each different fruit (counterbalanced order) and had a fixed response period. The results showed that 58.33% (14/24) of the infants in the Begging Experimenter Group picked up a fruit and gave it to the adult, and only 4.17% (1/24) of the infants in the Non-Begging Experimenter Group did so, χ^2^ (1, *N* = 48) = 13.96, *P* = 0.0002, ϕ = 0.58 (Fig. 1a) (all statistics in the paper are reported using two-tailed tests). The percent of trials in which the fruit was transferred was greater in the Begging Experimenter Group (*M* = 34.38%, *SD* = 39.57) than in the Non-Begging Experimenter Group (*M* = 4.17%, *SD* = 20.41), *z* = 3.02, *P* = 0.002, *r* = 0.44 (permutation test). The mean latency from infants’ securely grasping the fruits to transfer was 3.17 s (*SD* = 3.45). These results showed that human infants forgo nutrient-dense, natural foods for the sake of giving them to a stranger. Yet, one limitation of this result is that it is not clear that the infants interpreted the fruits as valuable for themselves. For this reason, we sought to examine whether infants also transferred the food after we had increased their motivation for the food such that it became advantageous for them to eat rather than to transfer the fruits.

### Experiment 2

**Figure 2:**
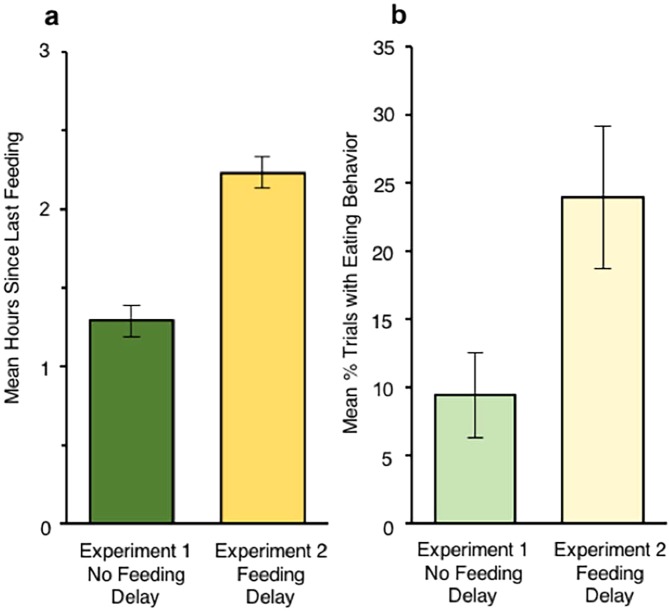
Delay in infant feeding and observed eating behavior. **(a)** Mean number of hours since infants’ last feeding in Experiments 1 and 2. **(b)** Mean percent of trials infants exhibited eating behavior during the test. Error bars show ±1 *SE*.

We designed this experiment to manipulate infants’ motivational state by asking parents to come to the laboratory immediately before the time when their infant would be ready for their next meal or snack, and before they fed them. This manipulation successfully increased infants’ time since last eating from Experiment 1 (*M* = 1.29 h, *SD* = 0.69) to Experiment 2 (*M* = 2.21 h, *SD* = 0.72), *z* = 5.35, *P* = 0.00000001, *r* = 0.55 (permutation test) (Fig. 2a). Not only was there a highly significant temporal delay in feeding time, but the experimental manipulation significantly changed infants’ eating behavior in the experiment itself. As shown in Fig. 2b, infants in Experiment 2 exhibited eating behavior on more trials (*M* = 23.96% of the trials, *SD* = 36.45) than infants in Experiment 1 (*M* = 9.38%, *SD* = 21.65), *z* = 2.33, *P* = 0.024, *r* = 0.24 (permutation test). The finding that infants in Experiment 2 engaged in more eating behavior than those in Experiment 1 shows that the infants understood that the fresh fruits were not common objects, but rather readily consumable.

The results show that 37.50% (9/24) of the infants in the Begging Experimenter Group transferred fruit compared to 0% (0/24) in the Non-Begging Experimenter Group, *P* = 0.0016, ϕ = 0.48, Fisher’s exact test, reported as a two-tailed test (Fig. 1b). The percent of trials with fruit transfer was significantly greater in the Begging Experimenter Group (*M = *29.17%, *SD* = 42.78) than in the Non-Begging Experimenter Group (*M = *0%), *z* = 3.03, *P* = 0.002, *r* = 0.44 (permutation test). Infants quickly transferred the fruit (*M* = 1.55 s, *SD* = 0.47). In sum, the experimental manipulation significantly lengthened the time since last feed and yet, as a group, infants still significantly transferred fruit to the stranger. We acknowledge that infants’ hunger at the individual level was not measured through blood glucose level or other assessments, which would be useful in drawing more precise inferences about “hunger” at an individual level.

### Pooled analyses

We combined the data across our two experiments and with this larger *N* analyzed additional issues of importance to theory. Below we report additional analyses which together provide a fuller picture of the nature and scope of human infant altruistic food transfer (see also Supplementary Information Note 3).

First, the main effects are strong: 47.92% (23/48) of the infants in the Begging Experimenter Group picked up a fruit and gave it to the adult, and only 2.08% (1/48) of the infants in the Non-Begging Experimenter Group did so, χ^2^ (1, *N* = 96) = 24.50, *P* = 0.0000007, ϕ = 0.53. The mean percent of trials with fruit transfer was greater in the Begging Experimenter Group (*M* = 31.77%, *SD* = 40.85) than in the Non-Begging Experimenter Group (2.08%, *SD* = 14.43), *z* = 4.29, *P* = 0.000004, *r* = 0.44 (permutation test). Infant performance on these measures did not significantly differ as a function of experiment (Supplementary Information Note 3).

Second, significantly more infants in the Begging Experimenter Group transferred the fruit on the *first trial* (14 of 48 infants; 6 from Experiment 1) than did the Non-Begging Experimenter Group (1 of 48), χ^2^ (1, *N* = 96) = 11.38, *P* = 0.0007, ϕ = 0.37, indicating that training or experience within the experiment was not necessary. Moreover, the same analyses on trials 2–4 showed that each of these trials was also significant: respectively, 0.00009, 0.0007, 0.0002. A Cochran *Q* test showed that infant fruit transfer did not significantly vary by trial, *Q*(3, *N* = 48) = 1.98, *P* = 0.58, η^2^_Q_ = 0.01.

**Figure 3:**
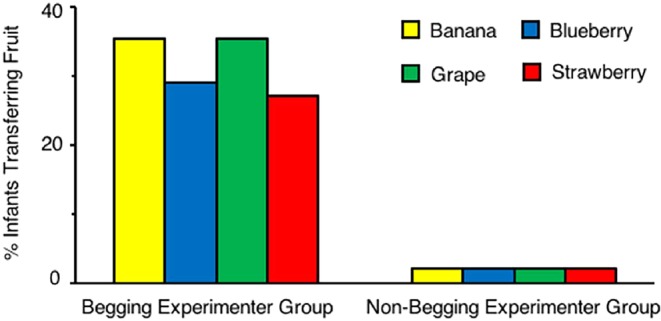
Transfers by type of fruit, across experiments. Percent of infants in each group transferring banana (yellow), blueberry (blue), grape (green), and strawberry (red).

Third, the transfer effect was generalizable across all tested fruits (Fig. 3): bananas, χ^2^ (1, *N* = 96) = 15.38, *P* = 0.00009, ϕ = 0.43; blueberries, χ^2^ (1, *N* = 96) = 11.38, *P* = 0.0007, ϕ = 0.37; grapes, χ^2^ (1, *N* = 96) = 15.38, *P* = 0.00009, ϕ = 0.43; strawberries, χ^2^ (1, *N* = 96) = 10.12, *P* = 0.002, ϕ = 0.35.

Fourth, infants readily transferred natural foods of high personal value and desirability. Parents rated infants’ liking of the fruits from 1 (“strongly dislike”) to 7 (“strongly like”) and the results showed that the individual fruits were highly-liked (*Mode* = 7, *M* = 5.99, *SD* = 0.74). Furthermore, parents reported on infants’ liking of vegetables (cucumber, peas, carrot, corn, and bell pepper), and the fruits were liked significantly more than the vegetables, *z* = 7.72, *P* = 10^−24^, *r* = 0.56 (permutation test).

Fifth, the psychological literature points to two aspects of social experience that are thought to influence social interactions even early in life: having siblings^29^ and being raised by parents from ethnic-cultural backgrounds that place particular value on the child’s connectedness to others in the group, which has been termed “interdependence” by social psychologists^26^. For example, Asian and Hispanic cultures are classified as high on interdependence^26,27,36^. Data obtained from parents about the presence of siblings and the ethnic-cultural background of the infants allowed us to explore the extent to which such social factors might be related to infants’ altruistic food transfer. We examined our data using a two-step hierarchical regression with the percent of trials with fruit transfer as the criterion. Experimental Group was entered as the predictor variable in Step 1 of the regression. We had no expectation for whether siblings or ethnic-cultural background would be more significant predictors, and thus entered them together as predictor variables in Step 2. The overall model explained 29.3% of the variance. Experimental Group accounted for 19.3% of the variance (*P* = 0.000007), and the socialization variables accounted for an additional 10% of the variance (*P* = 0.002), with both the ethnic-cultural background and sibling variables having significant positive effects (see Table 1). Although the regression model showed effects for these variables, we wish to express caution in interpreting these effects, because of the small sample size, the skewed sample of parents who come into research laboratories, and the desirability in the future of video recordings of infants’ sociocultural experiences at home. Direct experimental manipulations of family dynamics and/or social experiences would also be useful, because our findings may correlate with other notable demographics, such as socioeconomic status, which we did not collect. Nonetheless, the present data are consistent with previous work showing that infants from different cultures (e.g. Germany and India) vary in helping behavior^28^, as well as with work showing that having siblings is associated with cooperative behavior in toddlers^29^.

**Table 1:** Summary of hierarchical multiple regression analysis with percent of trials with food transfer as criterion (*N* = 96).

Regression step	Predictor variable	*b*	*SE*	*t*	Δ*R*^2^	*P*
Step 1	Group	29.69	6.25	4.75	0.19	0.000007
Step 2				2.54	0.10	0.002
	Siblings	13.83	6.18	2.24		0.028
	Ethnic-cultural background	30.77	10.21	3.02		0.003

Sixth, by design, the experimenter in the studies did not show reciprocity. The adult simply accepted the infants’ transfer of fruits and kept them for himself. (If infants picked up and transferred the fruit, the experimenter removed it from sight by depositing it in a container under the table and proceeded with the next trial.) Despite this lack of reciprocity, 10 of the 23 infants who transferred fruit in the Begging Experimenter Group (5 in each experiment) gave up the fruit on *every* trial. That is, for these particular infants it was a zero-sum interaction: They altruistically gave all of the food to the beggar, keeping nothing for themselves. This suggests that direct reciprocal exchanges with the adult during the experimental trials are not necessary to elicit or sustain the giving of desirable food, but this does not mean that reciprocity is unimportant in the evolution of altruism (see^11,37^ and Supplementary Information Note 4) or that reciprocal experiences within the culture, family, or other social situations prior to the experiment play no role (see fifth point above).

Seventh, we also checked two further points that provide useful material for interpreting the effects. (i) We checked whether the experimenter held onto the fruit for a similar duration in the experimental and control groups, and results indicated that this was the case: Begging Experimenter Group (*M = *6.81 s, *SD* = 2.26) and the Non-Begging Experimenter Group (*M* = 7.53 s, *SD* = 1.54), *z* = 1.79, *P* = 0.071, *r* = 0.18 (permutation test). This shows that the length of time the experimenter held onto the fruit before dropping it onto the tray was brief (about 7 s), and that this time of possession was roughly the same for both groups. (ii) We also confirmed that the number of infants engaging in eating behavior did not differ by group: 12 in Begging Experimenter Group versus 15 in Non-Begging Experimenter Group), χ^2^ (1, *N* = 96) = 0.21, *P* = 0.65, ϕ = −0.07. This result weighs against the possibility that infants may have only felt license to pick up and eat the food when the adult had intentionally discarded it (control group) and not when the experimenter merely “accidently” dropped it (experimental group).

## Discussion

Taken as a whole, the pattern of findings indicates that human infants systematically show altruistic food transfer behavior with readily edible, high-value food even when there is motivation to take desirous food for themselves (as in Experiment 2). This has not been documented in chimpanzees, although they clearly show social cooperation and other component prosocial skills^1,8–10^. In the classic study^9^ establishing that chimpanzees (human-raised, and regularly fed by humans) were capable of transferring dropped nonedible objects, the investigators noted that chimpanzees did not readily help with food—which is why the experimental test was designed to use nonedible items. The authors note: “It is possible that helping behaviors are more likely when they involve objects that are not food, and that this explains why we obtained positive results when others, using different tasks involving food, have found negative results” (p. 1302)^9^.

The direct transfer of high-value food shown in the current study is intriguing because of several aspects of the experimental design. First, infants were provided with a clear route for escaping with the food—the adult’s path to the infant was blocked by a table. Thus, infants could have retreated to their parent and eaten the food instead of giving it up. Second, we specifically used procedures in which the infant response was not linguistically scaffolded^25^, verbally requested^31^, directly modeled^35^, and the child was not explicitly thanked^30^ for sharing. As such, infants spontaneously overrode their biological drive for the desirable fruits and gave them to the non-kin beggar when the infants could have taken the food for themselves—and they did so on the first trial.

The overall pattern of findings not only underscores human infants’ capacity for this behavior but simultaneously suggests malleability. Consistent with prior work and theory holding that experiences build mental models of social interactions^38^, our findings invite the speculation that infants’ interactions in one context—such as with their parents^26,28^ or siblings^29^—may impact the expression of their helping tendencies toward a novel person in a laboratory context. Interestingly, formal mathematical models of human cooperation in adults are increasingly highlighting the value of incorporating factors such as family history, culture, and age in trying to account for individual-level variation in cooperation^39^.

There are also other experiences that could affect infants’ behavior in our experiments. For example, the feeding-delay manipulation in Experiment 2 at best provides an ethical analogue for exploring what infants might do in cases of severe food scarcity^4^ (we acknowledge that the behavior in Experiment 2 is not comparable to adult food altruism during famine, because the infants presumably had no reason to think that their current state would persist). It would be interesting to explore whether infants from a foraging society or infants from an industrialized society who have personal experience with chronic food insecurity also show the altruistic behavioral proclivities observed in the current work. Moreover, a history of short-term social interactions with new people—including responsive, positive social interactions with neighbors, doctors, or even a laboratory experimenter^40^—could help infants to build a mental representation of relationships involving trust and security^23,24^. Future research could be designed to examine the degree to which such social experiences modify the effects observed here.

The human readiness to actively engage in the observed behaviors raises issues about the possible functional significance of altruistic food transfer in human evolutionary history. By giving away food to strangers, individuals may promote dyadic affiliation and group cohesion^41^ and thereby species success within the dynamic environment of evolutionary adaptation^5,6^. Some other mammals, including *Callitrichidae*, *Canidae*, *Delphinidae*, as well as birds, have evolved food sharing behavior with kin, notably parental feeding of the young, but they do not readily or spontaneously engage in this activity with non-kin or strangers^1,9^. With this in mind, our studies were specifically designed so that the experimenter did not engage in the type of activities that would normally signal a kin relationship to a human child: The stranger was present in the child’s life for less than 20 minutes, there were no caregiving behaviors, no feeding, and essentially no touching at all (either between the experimenter and child or the experimenter and parent). As such, it appears unlikely that the infants interpreted their relationship to the experimenter in terms of kinship.

In sum, we found that human infants at 19 months of age, even when they are subject to a feeding delay in a novel context with a stranger, engage in spontaneous, robust, and repeated altruistic food transfer behavior of high-value, readily consumable natural foods. The developmental^1,24,40,42^, evolutionary^15,41,43,44^, and social-cognitive factors^22,45^ contributing to this altruistic activity deserve further continued study. We speculate that certain childrearing practices and values (e.g., a family environment that emphasizes the connectedness and commitment between self and others) convey the expectation to infants that people tend to help others^38^ and may engender in children a generalized feeling of interpersonal obligation towards other humans in need^24,40,42^. In this way, early social experiences in family settings can be understood as contributing to a psychological system that fuels the expression of humans’ altruistic potential.

## Methods

### Approval

The research was conducted with the approval of the Human Subjects Institutional Review Board (IRB) at the University of Washington, approval number: 00000832. All methods were performed in accordance with the relevant guidelines and regulations. All parents of the infant subjects provided informed consent.

### Participants

All subjects were healthy full-term infants recruited from the Seattle metropolitan area. By design, infants were tested within a narrow, 1-month age range in order to reduce variance due to age. Infants were recruited through a computerized infant subject pool maintained at the university. Soon after birth, parents were sent a postcard inviting them to participate in infant studies. Parents who returned the card were entered into the registry and later contacted by telephone and email to solicit participation. This pool also contained names of parents who registered through a web interface giving us permission to contact them for participation. Pre-established criteria used for recruiting infants into the experiments were: normal gestational age (±3 weeks of due date), typical birthweight (2.5–4.5 kg), and no medical or developmental problems according to parental report. Families received a nominal gift for participating and parking fees were reimbursed.

The final sample consisted of 96 infants ranging of 581 to 612 days. According to parental report, 81.25% of their children were White, 4.17% were Asian, and 14.58% were multiracial (indicated as more than one race), with 6.25% also reporting Hispanic or Latino ethnicity. Additional infants arrived at the university but were excluded using pre-established criteria. In Experiment 1, the exclusions occurred because of extreme infant fussiness-tiredness-sickness (*n* = 5), experimental-equipment problems (*n* = 9), and parental interference or infant refusal to give up clinging to parent (*n* = 3). In Experiment 2, the corresponding *n*s were 6, 9, and 4.

In Experiment 1, the 48 infants (24 boys, 24 girls) ranged from 581–612 days (*M* = 19.57 months, *SD* = 0.30). In Experiment 2, the 48 infants (24 boys, 24 girls) ranged from 581–611 days (*M* = 19.55 months, *SD* = 0.27 months). The sample size was preselected prior to the experiments. A power analysis (SAS software, Version 9.4) showed that a sample size of *n* = 24 per group would be sufficient in detecting reliable differences, assuming a large effect size (*d* = 0.80) at the 0.05 alpha level (two tailed) with a power of 0.80.

### Test environment, materials, and counterbalancing

The experiments took place in a quiet test room lined by blue curtains (270 cm × 190 cm). Three video cameras recorded the session for subsequent coding. The cameras were synched by the same time generator which labeled time on a frame-by-frame basis (30 frames/s). The experimenter camera provided a view of the table area and the experimenter’s body; the infant camera provided a view of the infant and the infant area, including the warmup area with the parent; the overview camera focused on a top-down view of the tray and the fruit.

Testing materials included the metallic tray onto which the fruit was dropped (38.1 cm × 27.9 cm × 6.5 cm), the demonstration table (152 cm × 50 cm × 45 cm) with a black cloth skirt so that the participants could not see underneath it, a gate to the side that the experimenter walked through to access the space behind the table and face the infant, and two blue cartons (38.5 cm × 38.5 cm × 78 cm) next to the table to divide the test room.

The order of fruit presentation was balanced within group in both experiments (*N* = 24 per group). There were four orders used, which resulted in 6 infants (3 boys and 3 girls) allocated to each order within each group. The four orders were chosen using a Latin square design so that each fruit occurred equally often in each of the four positions. Order 1 was banana, blueberry, grape, and strawberry; Order 2 was blueberry, banana, strawberry, and grape; Order 3 was grape, strawberry, banana, and blueberry; Order 4 was strawberry, grape, blueberry, and banana.

### Procedure

The design and procedure for the two experiments were the same, except for the instructions to parents in Experiment 2 to bring infants at a time in the day when their infant would be ready for their next meal or snack, and before they fed them.

Both experiments were conducted after performing informed consent in a separate waiting room. Following consent, the male experimenter walked the parent and infant to the testing room. Once there, the experimenter used a foot switch to turn on the cameras and said to the infant, “Look, there’s already some turtles waiting for us.” These small plastic turtles were visible near a bin of other toys that was covered with a cloth. The experimenter then asked the parent to sit in a chair. Parents were instructed to focus on a questionnaire for the remaining of the session. The experimenter then sat on the floor with the infant near the bin of toys.

Infants had the opportunity to warm up to the test room and experimenter by playing with the toys (pairs of small toy cows, lions, planes, puppies, turtles, whales, and large plastic rings). The warmup was brief (*M* = 5.48 mins, *SD* = 1.57) for both experiments. The experimenter encouraged the child to use the toys, commenting on each toy and showing particular actions that could be performed, such as squeezing the bath toys (lions, turtles, whales), shaking to produce a noise (plastic rings), and moving a particular toy part (cow head/leg/tail, dog head/feet, and plane propeller/wheels). The experimenter continued to allow the infant to play until the infant had seen all of the toys and touched at least one, after which the experimenter began to gather the toys back into the bin, announcing that the toys were going to sleep as he covered the bin.

For the test, the experimenter pulled a tray out from below the cloth skirt of the demonstration table. He then walked behind the table, closing the gate, so that he was on the other side of the table and blocked from the infant side. To call attention to the table, the experimenter placed a squeaky duck on the table, and after the infant looked at it, the experiment was launched. First, the experimenter drew a card from a container below the table which specified the infant’s random assignment to the experimental or control group. (The cards were managed by a separate experimenter to ensure that counterbalancing was achieved.) Next, the experimenter initiated the test phase by saying, “I’m going to show you some fruits I found, are you ready?”

Each infant was administered four trials, one with each fruit. Each trial consisted of the experimenter taking a piece of fruit from a storage bin hidden below the table and holding it up in front of him and excitedly naming the fruit to attract the infant’s attention (see verbal script below). From here the procedure branched to follow one of two paths according to the infant’s randomly assigned group.

In the Begging Experimenter Group, the experimenter fumbled the fruit such that it seemed to accidentally fall from his hand, landing out of his reach on to the tray on the floor in front of the table. A 20-s response period was timed from the moment the fruit hit the tray. In the first 10 s of the response period, the experimenter unsuccessfully reached for and looked to the fallen fruit; in the second 10 s, the experimenter continued to reach as he looked back and forth between the fruit and the infant. The 20-s response periods were electronically timed. The experimenter was notified when the response period expired via a silent vibrating timer. At the end of each trial, whether or not the infant handed him the fruit, the experimenter said “Ah, interesting.”

In the Non-Begging Experimenter Group, the experimenter also picked up a piece of fruit from beneath the table and named it to attract the infant’s attention, identically to the other group. He then appeared to intentionally aim and toss the fruit into the tray, whereupon he rested his hands on the table. The 20-s response period was electronically timed identically to the other group. As in the Begging Experimenter Group, at the end of each trial, whether or not the infant handed him the fruit, the experimenter said “Ah, interesting.”

The verbal script used by the experimenter when he initially picked up the fruit was identical for both groups. For the first trial the experimenter said, “It’s a banana, see the banana?” (The actual fruit names varied based on the counterbalanced order.) For the second trial he said, “Whoa, I found another fruit, it’s a blueberry, see the blueberry?” For the third trial he said, “Guess what, there’s another fruit, it’s a grape, see the grape?” Finally, for the fourth trial he said, “Oh my, there’s one more fruit! It’s a strawberry, see the strawberry?”

### Behavioral scoring

The infant behavior during the 20-s response period was scored from the video records, primarily based on the view from the experimenter camera facing the infant, but if the fruit was obscured by the infant, all camera angles were available to the coders to obtain the best view of the response. The coders were unaware of the experimental hypotheses and scored the infants in a random order. The coders scored whether or not the infant placed the fruit in the experimenter’s hand (transfer), engaged in eating behavior (lick, put in mouth, or swallow), and the latency to transfer the fruit for the infants who did so. The main coder scored all videos and a second coder scored a randomly selected 25% of the videos. Coders achieved excellent agreement on all measures as assessed by Cohen’s kappa. The mean interscorer agreement was *k* = 0.99; the mean intrascorer agreement was *k* = 0.99.

For the analysis of sibling and ethnic-cultural background variables, infants were classified according to parents’ responses on a paper-and-pencil questionnaire concerning the infants’ siblings, race, and ethnicity. Based on responses, infants were classified into those with and without siblings. The options provided for race and ethnicity followed guidelines from the National Institutes of Health^46^. Parents classified their infant as either Hispanic/Latino or not Hispanic/Latino ethnicity. In a separate item, parents indicated their infant’s race: American Indian/Alaskan Native, Asian, Black/African-American, Native Hawaiian/Other Pacific Islander, White. Work in social psychology^26,36,47,48^ has grouped Asians and Hispanics/Latinos as being from “interdependent cultures”; and we also grouped them together to derive the ethnic-cultural background variable.

### Preliminary analyses

Preliminary analyses in both experiments showed that none of the factors that were counterbalanced had a significant effect on the data. Specifically, there were no significant main effects or interactions based on sex of the infant or test order. Therefore, the main analyses are presented using the overall data.

## Supplementary information

Supplementary Information.

## Data Availability

The data that support the findings of these studies are available from the corresponding authors on reasonable request.
