# Supplementary material for: Altruistic food sharing behavior by human infants after a hunger manipulation
Source: Sci Rep. 2020 Feb 4;10:1785. doi: 10.1038/s41598-020-58645-9 (PMC7000707; doi:10.1038/s41598-020-58645-9)
Supplement: Supplementary file 1 — Supplementary Information. [file 41598_2020_58645_MOESM1_ESM.pdf]

Supplementary Information

Altruistic Food Sharing Behavior by Human Infants After a Hunger Manipulation

Scientific Reports doi: 10.1038/s41598-020-58645-9

Rodolfo Cortes Barragan[1,2*], Rechele Brooks[1], Andrew N. Meltzoff[1,2*]

[1]Institute for Learning & Brain Sciences, University of Washington

[2]Department of Psychology, University of Washington

*Correspondence to: barragan@uw.edu; meltzoff@uw.edu

Supplementary Note 1: Sharing Food Among Bonobos

Bonobos share food with others under certain experimental situations (for constraints, see [1] experiment 4). For example they are willing to open doors[1] and release ropes[2] to allow conspecifics access to food. The test of directly handing over high-value food in one's possession to a conspecific, while getting nothing in return, has received less attention. In the most seminal experiment to date with bonobos[3], investigators administered a pretest involving nuts that bonobos could crack open with rocks. If a subject passed this pretest, the experimenter moved the rocks to an adjacent cage that contained a second bonobo. Results showed that the first bonobo, who lacked the rocks for cracking open the nuts, moved the uncracked nuts closer to the conspecific and rocks: Specifically, "the possessor transferred [a nut] through the test window into the adjacent [conspecific's] room" (p. 4)[3]. Thus, bonobos moved nuts towards the conspecific and rocks, but this task did not involve transferring raw nut meat to the conspecific's cage (it involved the transfer of uncracked nuts, which are less desirable food). Importantly, as the authors note, "Bonobos appear to assign nuts intermediate value between high-value fruit and low-value foliage…," p. 3).

Another example derives from observations of bonobos in the wild. Male bonobos have been observed to allow females, including non-group members, to take pieces of meat from a carcass[4]. As authors pointed out, "the transfer of food from the male to the females was passive" (p. 96)[4]. Thus, this situation is closer to de Waal's ideas about "selective relinquishment" and passive sharing[5] from a pile of food (e.g., co-feeding) than to situations in which a single high-value food item is actively given away to another leaving nothing for oneself. The latter situation is common in human adults and also reported in the current paper with human infants.

The studies of food sharing in bonobos are of great value and rapidly advancing[6,7]. Continued research is desirable to demark the extent of and constraints on this behavior in the laboratory and in the wild.

Supplementary Note 2: Two Design Choices: Excluding Linguistic Support and Using Fruit

We made specific design choices in our study of food sharing in human infants. First, previous studies of infant food sharing used linguistic scaffolding (e.g. the adult verbally expressed a need by saying "I'm hungry, but I don't have anything to eat,")[8] and/or verbally thanked the infants for sharing food[9]. Second, these studies exclusively used manufactured foods (e.g. crackers and cereals), and we chose to use fruit—an evolutionarily salient type of resource[3,5]—in part because experimental studies of nonhuman primates' food sharing have often used fruit[1,2,10,11], facilitating comparison to those studies.

Supplementary Note 3: Further Statistical Analyses

For completeness, we conducted further analyses of the combined data from Experiments 1 and 2 based on the helpful suggestions of a reviewer. First, we used a generalized linear mixed model (GLMM) to predict the binary response of transferring fruit (yes/no per trial) using subject ID as a random effect, group as the main predictor, and trial, fruit type, experiment, and infant's sex as control variables that were assigned or counterbalanced by experimental design (SAS Version 9.4, proc glimmix with maximum likelihood estimation). As shown in Table S1, group was significant and none of the control variables was significant.

Table S1. Results from GLMM Predicting Infant Fruit Transfer

Predictor	*b*	*SE*	*F*	*P*
Group	10.52	3.03	12.03	0.0006
Trial	0.07	0.26	0.07	0.80
Fruit type	1.24	0.82	1.12	0.34
Experiment	1.73	1.64	1.12	0.29
Infant's sex	0.94	1.60	0.34	0.56

Note. $N = 96$.

We attempted to test the group × experiment interaction with GLMM, but because only one control infant across both experiments transferred fruit, that model did not converge, preventing a test of the interaction. However, several other analyses indicated that infant performance did not significantly vary as a function of experiment on the measures of interest. Using a chi-square test, we found the number of infants in the experimental group who transferred fruit in Experiment 1 (14/24) did not significantly differ from Experiment 2 (9/24), $\chi^2(1, N = 48) = 2.10$, $P = 0.15$. Similarly, using a permutation test, we found that the percent of trials with fruit transfer in the experimental group did not significantly differ between Experiment 1 ($M = 34.38$, $SD = 39.57$) and Experiment 2 ($M = 29.17$, $SD = 42.78$), $z = 0.44$, $P = 0.73$. Finally, using multiple regression and percent of trials with fruit transfer as the criterion, we were able to test the predictors of group, experiment, and their interaction (group × experiment). Results again showed that group was significant, $b = 30.21$ ($SE = 8.91$), $t(92) = 3.39$, $P = 0.001$; and the experiment and the group × experiment predictors were not significant: experiment, $b = -4.17$ ($SE = 8.91$), $t(92) = -0.47$, $P = 0.64$; group × experiment, $b = -1.04$ ($SE = 12.60$), $t(92) = -0.08$, $P = 0.93$.

Supplementary Note 4: Proximate and Ultimate Explanations of Altruism

We acknowledge that our experiments address a proximate-level[12] understanding of altruism. The findings suggest that direct reciprocity of the fruit exchanges with the experimenter is not needed within the test to elicit and maintain food transfers (see Discussion in the main text). This does not imply that food reciprocity is not important at the ultimate level[13]. Indeed, the "nutritional hypothesis"[14] theorizes that infants gain nutritional benefits from adults' food sharing, and that this may promote inclusive fitness or reproductive output[15,16] (suggesting that the ultimate function of food sharing could be described as "selfish"). Ultimate-level explanations for altruism are much debated[5,12] and beyond the focus of the current experiments concerning the proclivities of human infants.

References

1. Tan, J. & Hare, B. Bonobos share with strangers. *PLoS ONE* **8**, e51922 (2013).
2. Tan, J., Ariely, D. & Hare, B. Bonobos respond prosocially toward members of other groups. *Sci. Rep.* **7**, 14733 (2017).
3. Krupenye, C., Tan, J. & Hare, B. Bonobos voluntarily hand food to others but not toys or tools. *Proc. R. Soc. B* **285**, 20181536 (2018).
4. Fruth, B. & Hohmann, G. Food sharing across borders: First observation of intercommunity meat sharing by bonobos in LuiKotale, DRC. *Hum. Nat.* **29**, 91-103 (2018).
5. de Waal, F. B. M. Food sharing and reciprocal obligations among chimpanzees. *J. Hum. Evol.* **18**, 433-459 (1989).
6. Tan, J. & Hare, B. Prosociality among non-kin in bonobos and chimpanzees compared. In *Bonobos: Unique in Mind, Brain, and Behavior* (eds. Hare, B. & Yamamoto, S.) 140-154 (Oxford University Press, 2017).
7. Tomasello, M. *Becoming Human: A Theory of Ontogeny* (Harvard University Press, 2019).
8. Newton, E. K., Thomson, R. A. & Goodman, M. Individual differences in toddlers' prosociality: Experiences in early relationships explain variability in prosocial behavior. *Child Dev.* **87**, 1715-1726 (2016).
9. Dunfield, K., Kuhlmeier, V. A., O'Connell, L. & Kelley, E. Examining the diversity of prosocial behavior: Helping, sharing, and comforting in infancy. *Infancy* **16**, 227-247 (2011).
10. Silk, J. B. *et al.* Chimpanzees are indifferent to the welfare of unrelated group members. *Nature* **437**, 1357-1359 (2005).
11. Bullinger, A. F., Burkart, J. M., Melis, A. P. & Tomasello. M. Bonobos, *Pan paniscus,* chimpanzees, *Pan troglodytes,* and marmosets, *Callithrix jacchus,* prefer to feed alone. *Anim. Behav.* **85**, 51-60 (2013).
12. Preston, S. D. & de Waal, F. B. M. Empathy: Its ultimate and proximate bases. *Behav. Brain Sci.* **25**, 1-71 (2002).
13. Jaeggi, A. V. & Gurven, M. Reciprocity explains food sharing in humans and other primates independent of kin selection and tolerated scrounging: a phylogenetic meta-analysis. *Proc. R. Soc. B* **280**, 20131615 (2013).
14. Brown, G. R., Almond, R. E. A. & van Bergen, Y. Begging, stealing, and offering: Food transfer in nonhuman primates. *Adv. Study Behav.* **34**, 265-295 (2004).
15. Jaeggi, A. V. & Gurven, M. Natural cooperators: Food sharing in humans and other primates. *Evol. Anthropol.* **22**, 186-195 (2013).
16. Preston, S. D. The origins of altruism in offspring care. *Psychol. Bull.* **139**, 1305-1341 (2013).
